# Two-Dimensional Speckle Tracking Echocardiography Predict Left Ventricular Remodeling after Acute Myocardial Infarction in Patients with Preserved Ejection Fraction

**DOI:** 10.1371/journal.pone.0168109

**Published:** 2016-12-29

**Authors:** Ju-Feng Hsiao, Chang-Min Chung, Chi-Ming Chu, Yu-Shen Lin, Kuo-Li Pan, Shih-Tai Chang, Jen-Te Hsu

**Affiliations:** 1 The Department of Cardiology, Chiayi Chang Gung Memorial Hospital, Chang Gung University College of Medicine, Pu-Tz City, Chai Yi Hsien, Taiwan; 2 Section of Health Informatics, Institute of Public Health, National Defense Medical Center and University, Taipei, Taiwan; Scuola Superiore Sant'Anna, ITALY

## Abstract

**Objectives:**

Left ventricular remodeling after acute myocardial infarction increases cardiovascular events and mortality. But few study was done in patients with preserved ejection fraction (EF > 40%). We investigate whether the strain and strain rate by 2D speckle tracking echocardiography could predict left ventricular remodeling after acute myocardial infarction in this cohort.

**Methods:**

The 83 patients (average age 60.7 ± 12.3 y, 75 [90.4%] male) with new-onset acute myocardial infarction receiving echocardiography immediately, and 6 months after admission were grouped by the presence or absence of left ventricular remodeling. Strain and strain rate including longitudinal, circumferential, and radial direction were calculated. The average of strain and strain rate of which segmental longitudinal strains > – 15% were defined as the injury longitudinal strain (InjLS).

**Results:**

Left ventricular remodeling occurred in 24 of 83 patients (28.9%). In univariate logistic regression analyses, gender, peak CK-MB, log BNP, use of statin before discharge, wall motion score index, and InjLS were significantly associated with left ventricular remodeling (p < 0.05). In multivariate analysis using the forward stepwise method, gender, CK-MB, and InjLS were independent predictors. The hazard ratio for InjLS was 1.48 (p = 0.04). Receiver operating characteristic curve (ROC) analyses showed the area under the curve (AUC) of InjLS was largest (AUC = 0.75, cut-off value = –11.7%, sensitivity = 81%, specificity = 71%, p < 0.01). In ST-segment elevation myocardial infarction subgroup, InjLS was the only predictor according to ROC analysis (AUC = 0.79, p < 0.01, cut-off value = –11.4%, sensitivity = 88%, specificity = 77%) and multivariate logistic regression analysis (hazard ratio = 1.88, 95% CI: 1.22–2.88, p < 0.01).

**Conclusions:**

InjLS was an excellent predictor for left ventricular remodeling after acute myocardial infarction in patient with preserved ejection fraction.

## Introduction

Adverse left ventricular (LV) remodeling begins in some patients with acute myocardial infarction (AMI) even after percutaneous coronary intervention (PCI), and according to previous studies, the incidence is around 30%–35% [1–4]. LV remodeling leads to heart failure and increases the risks for cardiovascular events and mortality. Echocardiography is the first choice among imaging studies in patients with AMI. The left ventricular ejection fraction (LVEF) determined by conventional echocardiography and the wall motion score index (WMSI) have been reported as useful predictors for LV remodeling and clinical outcomes[1,5–7]. However, the prediction of WMSI in patients with preserved systolic heart function is uncertain [8,9]. Myocardial strain and strain rate measured by the 2D speckle tracking echocardiography can be used to evaluate myocardial performance and have been shown as a better tool to evaluate more subtle changes in LV function in many cardiac diseases. Several studies have used the 2D speckle tracking echocardiography to predict LV remodeling after ST-segment elevation myocardial infarction (STEMI) or after non–ST-segment elevation myocardial infarction (NSTEMI) [10–17], but no reports have investigated the role of myocardial strain and strain rate in patients with preserved ejection fraction (EF). Thus, the objective of this study was to evaluate whether myocardial strain and strain rate by 2D speckle tracking echocardiography predict adverse LV remodeling in patients with preserved EF following STEMI or NSTEMI.

## Materials and Methods

### Study population

From March 2010 to July 2013, we enrolled 94 patients who were admitted with new-onset AMI. Exclusion criteria included patients with severe valvular disease, atrial fibrillation or flutter, or history of myocardial infarction. Echocardiography was performed at baseline 3.2 ± 1.6 days after admission (2.7 ± 1.6 days after PCI), 3 months, and 6 months after AMI was diagnosed. This study was approved by the Ethics Committee of the Chiayi Chang Gung Memorial Hospital, and all patients provided written informed consent.

### Angioplasty protocols

Following the diagnosis of AMI, PCI was completed as soon as possible. The average of door-to-balloon time for STEMI patients was 122 ± 289 min (median = 71 min) and for NSTEMI patients was 1937 ± 1864 min (median = 1417 min). PCI was considered successful if the residual stenosis was < 30% and the flow in the culprit vessel was ≥ Grade 2 according to the Thrombolysis in Myocardial Infarction (TIMI) score. The diseased vessel was defined as ≥ 50% stenosis. Findings of coronary angiography including culprit vessel, diseased vessels, left main involvement, single or multi-vessels (≥ 2 vessels) were recorded.

### Echocardiography

Comprehensive 2D transthoracic grayscale echocardiography was performed using a GE Vivid 7 echocardiographic system (M3S probe, Vivid 7, GE Vingmed, Horten, Norway). Images of 3 consecutive cardiac cycles in 3 apical views and short-axis views were stored digitally for off-line analysis with EchoPAC, version 11.0 (GE Vingmed). Frame rate of these images were 66–79 frames/s. LV and atrial volume, wall motion, and EF were assessed. LV end-diastolic diameter and end-systolic diameter were calculated according to the American Society of Echocardiography recommendations [18,19]. Stroke volume was determined by Doppler echocardiography and then was indexed by body surface area to facilitate derivation of stroke volume index. LVEF and LV volume were calculated by the modified Simpson's biplane method. Regional wall motion was visually evaluated with a 17-segment model in which each segment was scored as: 1 = normal, 2 = hypokinesia, 3 = akinesia, 4 = dyskinesia, and 5 = aneurysmal change. The WMSI was averaged from scores of the evaluated segments [18].

Peak early (E) and late diastolic wave velocity (A) were measured by pulse-wave velocity, and tissue Doppler imaging measured peak early (e′) and late (a′) diastolic velocity at the mitral septal annulus [19].

LV remodeling was defined as a > 15% increase in biplane LV end-systolic volume from the initial presentation to the 6-month follow-up. The population was divided into 2 groups: the remodeling group and nonremodeling group. Preserved EF was defined as EF > 40%.

### Speckle-tracking technique for measuring LV deformation performance

**Fig 1:**
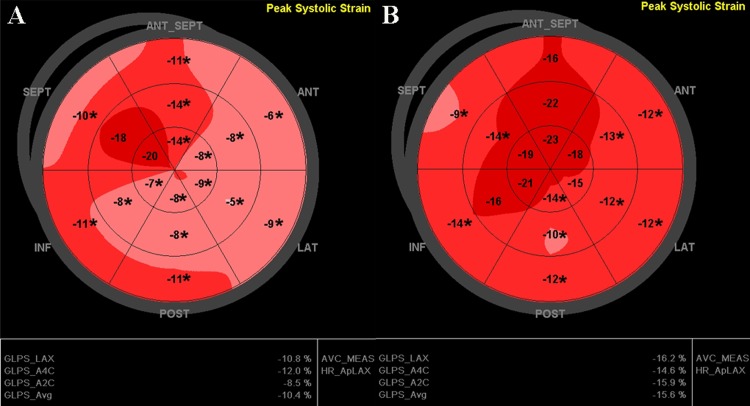
Illustration of injury longitudinal strain (A) A case with left ventricular remodeling; injury longitudinal strain is –9.2%. (B) A case without left ventricular remodeling: injury longitudinal strain is –12.2%. Abnormal segments are marked with *, which indicates that the longitudinal strain is >–15%.

The stored grayscale images were analyzed off-line to measure LV deformation performance via the 2D speckle-tracking technique (EchoPAC version 11.0). Global and segmental strain and strain rate in 3 directions (longitudinal, circumferential, and radial) were calculated. The LV radial and circumferential strains and strain rates were determined from the short-axis views at the basal, middle, and apical levels, and longitudinal strains and strain rates were determined from the apical 2-, 3-, and 4-chamber views of the LV. We traced the endocardial border manually at end-systole and adjusted width of the region of interest to cover the entire myocardium. The software then automatically tracked the myocardium. Poor tracking quality was revised manually until the quality was acceptable. Each apical view or short-axis view was divided into 6 segments and there were 18 segments in total. The global peak longitudinal strain (GLS), global peak circumferential strain (GCS), and radial strain (GRS) were averaged from the total 18 segments. Systolic strain rates were analyzed for longitudinal systolic strain rates (LSRs), circumferential systolic strain rates (CSRs), and radial systolic strain rates (RSRs). According to previous studies, segmental longitudinal strains > – 15% were defined as injured segments [20,21]. The average segmental longitudinal strain and strain rate of the abnormal segments was defined as the injury longitudinal strain (InjLS) (Fig 1) and injury longitudinal systolic strain rate (InjLSRs). The number of injured segments was also recorded. The average longitudinal strains and strain rates of territories of the culprit vessels were also calculated as culprit longitudinal strain (culprit LS) and culprit longitudinal strain rate (culprit LSRs).

### Serum biomarkers

Biochemical tests during hospitalization included serum creatinine level, high-sensitivity C-reactive protein (hs-CRP) and brain natriuretic peptide (BNP). Serial creatine kinase MB isoenzymes (CK-MB) were collected immediately and after 8 hours and 16 hours. Estimated glomerular rate (eGFR) was measured from 4-variable Modification of Diet in Renal Disease (MDRD) formula

### Statistical analysis

Continuous variables are expressed as means ± standard deviations for variables with normal distribution and as median (25^th^, 75^th^ percentile) for variables that were not normally distributed by Kolmogorov–Smirnov tests. Categorical variables are presented as the numbers of subjects and percentages. We used SPSS 21 (SPSS, Chicago, IL, USA) for statistical analyses. For comparisons between the 2 study groups, two-tailed Student’s *t*-test was used for continuous normal-distributed variables and Mann-Whitney U test was used for non-normal-distributed variables, and chi-squared tests were used for categorical variables. Binary logistic regression analysis was used to assess the predictors for LV remodeling. Significant variables with a p value < 0.1 by univariate analyses underwent further multivariate analyses using a forward stepwise logistic regression model. A p value < 0.05 was considered significant. A receiver operating characteristic curve (ROC) analysis was performed to assess the abilities of GLS, InjLS, InjLSRs, EF, and WMSI to predict LV remodeling and to identify the optimal cut-off value with optimal sensitivity and specificity. A p value < 0.05 was regarded as significant.

Ten subjects were randomly selected to assess the inter- and intra-observer variability. All LV deformation performance indices were measured by two independent observers for inter-observer variability. The same observer repeated the measurements 1 month later for intra-observer variability. The variability which was expressed as percentages was derived as the absolute difference between the 2 sets of measurements, divided by the overall mean of the 2 sets of measurements.

## Results

Ninety four patients were enrolled initially. Four patients were excluded due to EF ≤ 40%. During 6-month follow-up, 2 patients had expired, 2 patient receiving CABG dropped out due to poor echocardiogram images and 3 patients dropped out due to personal reasons. Thus, these 7 patients’ echocardiograms were not included in the calculation of LV remodeling. Five patients received new coronary angioplasty. Our final population included 83 patients (average age 60.7 ± 12.3 y, 75 [90.4%] male). LV remodeling occurred in 24 of 83 patients (28.9%). Table 1 compares the clinical characteristics of these 2 groups. In the remodeling group, peak CK-MB and the percentage of females were significantly higher than in the nonremodeling group. In subgroup of STEMI, the door-to-balloon time was longer in the remodeling group. The proportion of patients with STEMI was higher in the remodeling group than in the nonremodeling group, but this finding was not statistically significant (STEMI vs. NSTEMI = 79.2% vs. 20.8%, p = 0.09). The incidences of LV remodeling in STEMI and NSTEMI were 35.2% and 16.7%, respectively. The distributions of culprit vessels and single- or multi-vessel lesions were similar. The proportions of left anterior descending artery (LAD) to non–left anterior descending artery were 52.5% in the nonremodeling group and 37.5% in the remodeling group, respectively (p = 0.43). The PCI success rate was 100%, and the TIMI flow grade was 3 after PCI. There was no significant difference in Killip classes between these 2 groups.

**Table 1:** Patients’ clinical characteristics.

	No remodeling (N = 59)	Remodeling (N = 24)	p Value
Age (y)	59.4 ± 12.2	64.5 ± 11.4	0.08
Male sex	57 (96.6%)	18 (75%)	< 0.01*
hsCRP (mg/L)‡	13.4 (5.8, 41.3)	30.9 (5.0, 48.4)	0.56
Peak CK-MB (ng/mL)‡	33.6 (5.6, 117.8)	125.4 (31.7, 300)	0.01*
BNP (ng/L)‡	169 (62.3, 312)	206.5 (139.8, 520.8)	0.07
eGFR (mL/min/1.73 m^2^)	80.8 ± 26.3	72.4 ± 26.0	0.19
STEMI/NSTEMI	35 (59.3%)/24 (40.7%)	19 (79.2%)/5 (20.8%)	0.09
D-to-B (min)‡			
• 2022STEMI	67 (54, 76.5)	82 (71, 103)	0.01*
• NSTEMI	1458 (614, 3580)	853 (78, 2035)	0.15
S-to-B (h)‡			
• STEMI	3.5 (2.3, 6.0)	3.5 (2.7, 6.8)	0.68
• NSTEMI	32 (22, 81)	17 (9.9, 65)	0.39
Hypertension	36 (61%)	13 (54.2%)	0.57
Diabetes mellitus	15 (25.4%)	7 (29.2%)	0.73
Smoking	34 (57.6%)	14 (58.3%)	0.95
Medication before discharge			
ACEI or ARB	25 (42.4%)	11 (45.8%)	0.77
Beta-blocker	31 (52.5%)	14 (58.3%)	0.63
Statin	45 (76.3%)	13 (54.2%)	0.05*
Culprit vessel			
• LAD	31 (52.5%)	9 (37.5%)	0.43
• LCX	7 (11.9%)	(12.5%)	
• RCA	21 (35.6%)	12 (50%)	
Coronary artery disease			
• 1-vessel disease	19 (32.2%)	7 (29.2%)	0.81
• 2-vessel disease	25 (42.4%)	50%)	
• 3-vessel disease	15 (25.4%)	5 (20.8%)	
Diseased site			
• LM	7 (11.5%)	2 (8.3%)	1.0
• LAD	53 (89.8%)	20 (83.3%)	0.46
• LCX	25 (42.4%)	9 (37.5%)	0.68
• RCA	36 (61%)	17 (70.8%)	0.40
Killip class			0.42
I	41 (69.5%)	20 (83.3%)	
II	6 (10.2%)	1 (4.2%)	
III	4 (6.8%)	0	
IV	8 (13.6%)	3 (12.5%)	

* = p Vale <0.05

‡variables that were not normally distributed were expressed as median (25^th^, 75^th^ percentile); ACEI = angiotensin converting enzyme inhibitor; ARB = angiotensin receptor blocker; BNP = brain natriuretic peptide; CK-MB = creatine kinase MB isoenzyme; D-to-B = door-to-balloon time; eGFR = estimated glomerular filtration rate; HsCRP = high-sensitivity C-reactive protein; LAD = left anterior descending artery; LCX = left circumflex artery; LM = left main; NSTEMI = non–ST-segment elevation myocardial infarction; RCA = right coronary artery; S-to-B = symptom-onset-to-balloon time; STEMI = ST-segment elevation myocardial infarction.

Table 2 displays echocardiographic findings. In the remodeling group, the initial LV end-diastolic and end-systolic volumes were smaller and end-systolic volume at 6^th^ month follow-up became larger. There was no difference in EF, stroke volume index, left atrial volume index, E/A ratio, and E/e′ ratio. WMSI was higher in LV remodeling group (p = 0.03).

**Table 2:** Echocardiography findings.

	Nonremodeling (N = 59)	Remodeling (N = 24)	p Value
Baseline echocardiography after admission (days)	3.3 ± 1.5	3.1 ± 2.0	0.58
Baseline echocardiography after PCI (days)	2.6 ± 1.4	2.9 ± 2.0	0.44
Heart rate (baseline) (bpm)	72 ± 11	75 ± 9	0.31
Heart rate (6^th^ month) (bpm)	71 ± 12	68 ± 10	0.32
Ejection fraction (baseline) (%)	58.1 ± 7.3	59.3 ± 10.5	0.62
Ejection fraction (6^th^ month) (%)	63.9 ± 7.9	56.3 ± 8.0	<0.01*
Left ventricular end-diastolic volume (baseline) (mL)	111 ± 28	86 ± 24	< 0.01*
Left ventricular end-diastolic volume (6^th^ month) (mL)	107 ± 26	117 ± 34	0.23
Left ventricular end-systolic volume (baseline) (mL)	47 ± 15	35 ± 13	< 0.01*
Left ventricular end-systolic volume (6^th^ months) (mL)	39 ± 14	52 ± 23	0.02*
Stroke volume index (mL/m^2^)	40 ± 10.7	38.6 ± 12.4	0.63
Left atrial volume index (mL/m^2^)	32.6 ± 10.6	34.1 ± 11.1	0.61
Mitral inflow			
• E (cm/s)	82.4 ± 18.6	85.3 ± 17.4	0.52
• A (cm/sec)	81.2 ± 23.2	89.2 ± 30.1	0.2
• E/A	1.11 ± 0.44	1.08 ± 0.47	0.78
Deceleration time (ms)	199 ± 63	201 ± 63	0.91
Tissue Doppler image			
• E/e′	14.6 ± 5.1	16.6 ± 5.9	0.12
WMSI	1.26 ± 0.21	1.38 ± 0.25	0.03*

* = p Vale <0.05; A = late diastolic wave velocity; a′ = late diastolic wave velocity; E = peak early wave velocity; e′ = peak early diastolic velocity at mitral septum; s′ = systolic velocity at mitral septum by tissue Doppler image; WMSI = wall motion score index.

**Fig 2:**
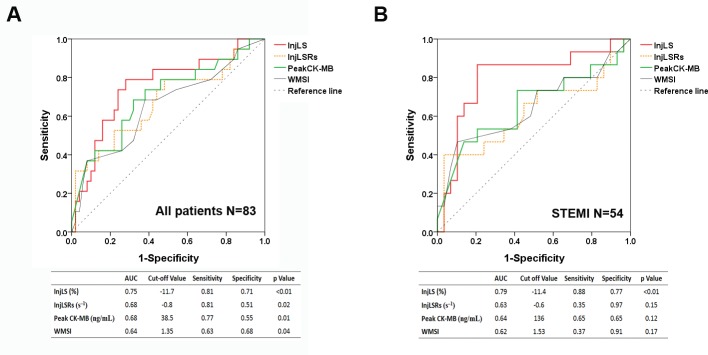
Receiver operating curve analysis for the prediction of left ventricular remodeling 6 months after acute myocardial infarction (A) for all population (B) for a subgroup of ST-segment elevation myocardial infarction patients.

Table 3 shows the LV deformation performance indices at baseline and 6^th^ month. At baseline, only InjLS and InjLSRs were significantly worse in the remodeling group. Number of injured segments was not different. ROC analyses (Fig 2A) were performed to evaluate the ability of peak CK-MB, WMSI, InjLS and InjLSRs to differentiate LV remodeling from nonremodeling. The area under the curve (AUC) of InjLS was largest (AUC = 0.75, p < 0.01) than the others (Fig 2). The optimal cut-off value of InjLS was –11.7% with 81% sensitivity and 71% specificity.

**Table 3:** Left ventricular deformation performance indices.

	Nonremodeling(N = 59)	Remodeling (N = 24)	p Value
**Baseline value**			
Global longitudinal strain (%)	–18.0 ± 3.6	–17.0 ± 4.2	0.3
Global longitudinal systolic strain rate (s^–1^)	–1.10 ± 0.21	–1.07 ± 0.23	0.5
Global circumferential strain (%)	–17.6 ± 4.2	–17.6 ± 5.0	0.96
Global circumferential systolic strain rate (s^–1^)	–1.44 ± 0.39	–1.46 ± 0.38	0.9
Global radial strain (%)	36.0 ± 12.5	37.5 ± 12.3	0.66
Global radial systolic strain rate (s^–1^)	1.8 ± 0.43	1.74 ± 0.32	0.61
Culprit longitudinal strain (%)	–16.7 ± 4.2	–15.5 ± 5.3	0.28
Culprit longitudinal systolic strain rate (s^–1^)	–1.00 ± 0.24	–0.96 ± 0.3	0.51
Injury longitudinal strain (%)	–12.4 ± 1.6	–10.9 ± 1.7	<0.01*
Injury longitudinal s systolic strain rate (s^–1^)	–0.81 ± 0.15	–0.71 ± 0.15	0.02*
No of injured segments	5.3 ± 4.8	6.8 ± 5.5	0.23
**6^th^ month value**			
Global longitudinal strain (%)	-19.6 ± 3.0	-18.2 ± 4.0	0.1
Global longitudinal systolic strain rate (s^–1^)	-1.15 ± 0.19	-0.97 ± 0.22	<0.01*
Global circumferential strain (%)	-19.4 ± 4.5	-17.9 ± 3.9	0.25
Global circumferential systolic strain rate (s^–1^)	-1.46 ± 0.39	-1.34 ± 0.26	0.26
Global radial strain (%)	39.3 ± 14.5	29.7 ± 15.3	0.03*
Global radial systolic strain rate (s^–1^)	1.76 ± 0.43	1.44 ± 0.43	0.02*
Culprit longitudinal strain (%)	-19.1 ± 3.6	-17.2 ± 4.7	0.07
Culprit longitudinal systolic strain rate (s^–1^)	-1.11 ± 0.23	-0.9 ± 0.29	<0.01*
Injury longitudinal strain (%)	-16.6 ± 3.19	-13.9 ± 3.0	<0.01*
Injury longitudinal systolic strain rate (s^–1^)	-1.0 ± 0.24	-0.75 ± 0.25	<0.01*

* = p Vale <0.05

Results of univariate logistic regression analyses to access the association with LV remodeling are shown in Table 4. BNP and hsCRP were log-transformed because they were not normal distributed. Sex, peak CK-MB, use of statin before discharge, log BNP, WMSI, InjLS and InjLSRs were significantly associated with LV remodeling (p < 0.05). Variables with p value < 0.1 by univariate analyses were submitted for multivariate analyses and included age, sex, peak CK-MB, use of statin, log BNP, WMSI, InjLS and InjLSRs. In multivariate analysis using the forward stepwise method, gender, CK-MB, and InjLS were independent predictors of LV remodeling in patients with AMI. The hazard ratio for CK-MB was 1.01 (p = 0.05) and for InjLS was 1.48 (p = 0.04).

**Table 4:** Univariate and multivariate logistic regression for left ventricular remodeling.

	Univariate analysis odds ratio (95% CI)	p Value	Multivariate analysis odds ratio (95% CI)	p Value
Age (y)	1.04 (1.0–1.08)	0.09		
Male sex	0.11 (0.02–0.57)	0.01*	0.11 (0.02–0.79)	0.03*
Peak CK-MB (ng/mL)	1.01 (1.00–1.01)	0.01*	1.01 (1.0–1.01)	0.05*
log BNP (ng/L)	2.89 (1.06–7.88)	0.04*		
log hsCRP (mg/L)	1.2 (0.54–2.67)	0.66		
eGFR (mL/min/1.73 m^2^)	0.99 (0.97–1.01)	0.19		
LAD disease	1.77 (0.45–6.92)	0.41		
LAD culprit lesion	1.85 (0.7–4.88)	0.22		
D-to-B (min)				
• STEMI	1.0 (0.99–1.00)	0.59		
• NSTEMI	1.0 (0.99–1.00)	0.26		
S-to-B (h)				
• STEMI	0.98 (0.93–1.04)	0.54		
• NSTEMI	0.99 (0.96–1.02)	0.41		
ACEI/ARB	0.87 (0.36–2.26)	0.77		
Beta-blocker	0.79 (0.30–2.05)	0.63		
Statin	2.72 (1.0–7.41)	0.05*		
Ejection fraction (%)	1.02 (0.96–1.08)	0.55		
WMSI	9.99 (1.15–87)	0.04*		
Left atrial volume index (mL/m^2^)	1.01 (0.97–1.06)	0.60		
Global longitudinal strain (%)	1.07 (0.94–1.2)	0.29		
Global longitudinal systolic strain rate (s^–1^)	2.02 (0.23–17.8)	0.53		
Global circumferential strain (%)	1.00 (0.89–1.13)	0.96		
Global circumferential systolic strain rate (s^–1^)	0.9 (0.23–3.49)	0.87		
Global radial strain (%)	1.01 (0.97–1.05)	0.65		
Global radial systolic strain rate (s^–1^)	0.7 (0.19–2.66)	0.6		
Injury longitudinal strain (%)	1.7 (1.21–2.39)	< 0.01*	1.48 (1.02–2.14)	0.04*
Injury longitudinal systolic strain rate (s^–1^)	112.3 (1.93–6541.2)	0.02*		
Culprit longitudinal strain (%)	1.06 (0.95–1.18)	0.27		
Culprit longitudinal systolic strain rate (s^–1^)	1.88 (0.29–12.2)	0.51		

* = p Vale <0.05; for acronym key, see Table 1.

In subgroup analysis for STEMI patients (Tables 5–7), the percentage of females and BNP were higher and the door-to-balloon time was longer in the remodeling group. Higher peak CK-MB trended toward LV remodeling but did not reach statistical significance. The proportion of culprit vessels on LAD and multi-vessel diseases were similar. In the LV remodeling group, the initial LV end-systolic or diastolic volume was smaller and end-systolic volume became larger at 6^th^ month follow-up. Only InjLS was different from non–LV remodeling among LV deformation performance indices.

**Table 5:** Clinical data for STEMI patients.

	Nonremodeling (N = 35)	Remodeling (N = 19)	p Value
Age (y)	58.3 ± 12.5	64.6 ± 11.2	0.07
Male sex	34 (97.1%)	15 (78.9%)	0.05*
hsCRP(mg/L)‡	18.4 (6.2, 56.7)	38 (7.6, 68.6)	0.32
Peak CK-MB (ng/mL)‡	58.8 (19.1, 216.3)	165(24.5, 300)	0.12
BNP (ng/L)‡	207 (63, 207)	343 (145, 844)	0.03*
eGFR (mL/min/1.73 m^2^)	84.8 ± 28.1	74.6 ± 26.6	0.2
D-to-B (min)‡	67 (54, 76.5)	82 (71, 103)	0.01*
S-to-B (h)‡	3.5 (2.3, 6.0)	3.5 (2.7, 6.8)	0.68
Hypertension	17 (48.6%)	9 (47.4%)	0.93
Diabetes mellitus	7 (20.0%)	6 (31.6%)	0.34
Smoking	24 (68.6%)	10 (52.6%)	0.25
**Medication before discharge**			
ACEI or ARB	15 (42.9%)	9 (47.4%)	0.75
Beta-blocker	20 (57.1%)	12 (63.2%)	0.67
Statin	28 (80%)	11 (57.9%)	0.08
**Culprit Vessel**			0.79
• LAD	18 (51.4%)	(42.1%)	
• LCX	2 (5.7%)	(5.3%)	
• RCA	15 (42.9%)	10 (52.6%)	
**Coronary artery disease**			0.8
• 1-vessel disease	11 (31.4%)	(31.6%)	
• 2-vessel disease	18 (51.4%)	57.9%)	
• 3-vessel disease	6 (17.1%)	2 (10.5%)	
**Diseased site**			
• Left main	4 (11.4%)	1 (5.3%)	0.65
• LAD	31 (88.6%)	16 (84.2%)	0.65
• LCX	13 (37.1%)	4 (21.1%)	0.36
• RCA	21 (60%)	14 (73.7%)	0.38

* = p Vale <0.05

‡variables that were not normally distributed were expressed as median (25^th^, 75^th^ percentile), for acronym key, see Table 1.

**Table 6:** Echocardiographic findings in STEMI patients.

	Nonremodeling (N = 35)	Remodeling (N = 19)	p Value
Baseline echocardiography after admission (days)	2.9 ± 1.4	3.3 ± 2.1	0.4
Baseline echocardiography after PCI (days)	2.8 ± 1.4	3.3 ± 2.0	0.37
Heart rate (baseline) (bpm)	72 ± 16	77 ± 17	0.36
Heart rate (6^th^ month) (bpm)	72 ± 12	69 ± 11	0.43
Ejection fraction (baseline) (%)	56.6 ± 7.9	57.6 ± 11.3	0.7
Ejection fraction (6^th^ month) (%)	62.8 ± 9.1	54.5 ± 7.9	<0.01*
Left ventricular end-diastolic volume (baseline) (mL)	116 ± 29	88 ± 25	< 0.01*
Left ventricular end-diastolic volume (6^th^ month) (mL)	111 ± 25	121 ± 37	0.31
Left ventricular end-systolic volume (baseline) (mL)	51 ± 16	37 ± 14	< 0.01*
Left ventricular end-systolic volume (6^th^ months) (mL)	42 ± 15	56 ± 24	<0.01*
Stroke volume index (mL/m^2^)	39.6 ± 11.7	35 ± 10.7	0.18
Left atrial volume index (mL/m^2^)	33.3 ± 8.3	34.9 ± 11.6	0.59
Mitral inflow			
• E (cm/s)	82.6 ± 18.0	86.0 ± 19.1	0.52
• A (cm/s)	79.1 ± 24.1	85 ± 31.3	0.45
• E/A	1.15 ± 0.47	1.14 ± 0.5	0.97
Deceleration time (ms)	204.6 ± 71.7	194.2 ± 66.2	0.62
Tissue Doppler image			
• E/e′	14.27 ± 5.14	16.56 ± 6.05	0.16
WMSI	1.31 ± 0.19	1.42 ± 0.26	0.1

* = p Vale <0.05; for acronym key, see Table 2.

**Table 7:** Left ventricle deformation performance indices in STEMI patients.

	Nonremodeling (N = 35)	Remodeling (N = 19)	p Value
**Baseline value**			
Global longitudinal strain (%)	–17.4 ± 3.7	–16.2 ± 4.3	0.27
Global longitudinal systolic strain rate(s^–1^)	–1.06 ± 0.2	–1.06 ± 0.26	0.99
Global circumferential strain (%)	–16.5 ± 3.9	–16.7 ± 4.8	0.9
Global circumferential systolic strain rate (s^–1^)	–1.34 ± 0.29	–1.4 ± 0.35	0.53
Global radial strain (%)	35.6 ± 14.3	35.7 ± 12.2	0.98
Global radial systolic strain rate (s^–1^)	1.76 ± 0.48	1.7 ± 0.29	0.70
Culprit longitudinal strain (%)	–16.1 ± 4.0	–14.5 ± 5.4	0.21
Culprit longitudinal systolic strain rate (s^–1^)	–0.96 ± 0.21	0.93 ± 0.31	0.73
Injury longitudinal strain (%)	–12.3 ± 1.7	–10.5 ± 1.6	< 0.01*
Injury longitudinal systolic strain rate (s^–1^)	–0.79 ± 0.13	–0.72 ± 0.16	0.13
No of injured segments	5.8 ± 5.1	8.1 ± 5.5	0.14
**6**^**th**^ **month value**			
Global longitudinal strain (%)	-18.9 ± 3.1	-17.6 ± 4.2	0.23
Global longitudinal systolic strain rate (s^–1^)	-1.1 ± 0.2	-0.9 ± 0.2	0.02*
Global circumferential strain (%)	-18.6 ± 4.8	-17.5 ± 4.0	0.51
Global circumferential systolic strain rate (s^–1^)	-1.3 ± 0.4	-1.3 ± 0.2	0.72
Global radial strain (%)	38.7 ± 14.2	25.5 ± 12.5	0.01*
Global radial systolic strain rate (s^–1^)	1.8 ± 0.5	1.3 ± 0.3	0.01*
Culprit longitudinal strain (%)	-18.4 ± 3.9	-16.4 ± 4.8	0.13
Culprit longitudinal systolic strain rate (s^–1^)	-1.1 ± 0.2	-0.9 ± 0.3	0.02*
Injury longitudinal strain (%)	-16.3 ± 3.4	-13.6 ± 3.0	0.02*
Injury longitudinal systolic strain rate (s^–1^)	-1.0 ± 0.3	-0.7 ± 0.3	<0.01*

* = p Vale <0.05

ROC analysis revealed that only InjLS was a significant predictor of LV remodeling (AUC = 0.79, p < 0.01, cut-off value –11.4%, sensitivity 88%, specificity 77%) (Fig 2B). Table 8 displays the results of logistic regression analyses. BNP and hsCRP were also log-transformed. In univariate logistic regression analysis, InjLS and log BNP were significant (p < 0.05). In multivariate analysis with the forward stepwise method, only InjLS was the only one predictors of LV remodeling in STEMI patients (hazard ratio = 1.88, 95% CI: 1.22–2.88, p < 0.01).

**Table 8:** Univariate logistic regression for left ventricular remodeling in STEMI patients.

	Univariate analysis odds ratio (95% CI)	p Value
Age	1.05 (0.99–1.102)	0.08
Male sex	0.11 (0.01–1.07)	0.06
Peak CK-MB (ng/mL)	1.01 (1.00–1.01)	0.06
log BNP (ng/L)	4.41 (1.19–16.28)	0.03*
log hsCRP (mg/L)	1.46 (0.53–4.04)	0.47
eGFR (mL/min/1.73 m^2^)	0.99 (0.97–1.01)	0.2
LAD disease	1.45 (0.29–7.3)	0.65
LAD culprit lesion	0.69 (0.22–2.12)	0.51
CAD number	0.86 (0.37–2.00)	0.72
D-to-B (min)	1.0 (0.99–1.00)	0.59
S-to-B (h)	0.98 (0.93–1.04)	0.54
ACEI/ARB	1.20 (0.39–3.69)	0.75
Beta-blocker	1.29 (0.41–4.05)	0.67
Statin	0.34 (0.1–1.18)	0.09
Ejection fraction (%)	1.01 (0.95–1.08)	0.71
WMSI	9.16 (0.63–133.0)	0.11
Left atrial volume index (mL/m^2^)	1.02 (0.96–1.09)	0.59
Global longitudinal strain (%)	1.09 (0.94–1.26)	0.26
Global longitudinal strain rate (s^-1^)	0.98(0.07–13.04)	0.99
Global circumferential strain (%)	0.99 (0.85–1.15)	0.9
Global circumferential strain rate (s^-1^)	0.51(0.07–3.97)	0.52
Global radial strain (%)	1.00 (0.96–1.05)	0.98
Global radial strain rate (s^-1^)	0.74 (0.17–3.276)	0.7
Injury longitudinal strain (%)	1.88 (1.22–2.88)	< 0.01*
Injury longitudinal strain rate (s^-1^)	28.72(0.35–2390.8)	0.14
Culprit longitudinal strain (%)	1.09 (0.96–1.23)	0.21
Culprit longitudinal strain rate (s^-1^)	1.6(0.16–16.11)	0.69

* = p Vale <0.05; for acronym key, see Table 1.

Intra-observer variability for GLS, GCS and GRS was 3.4 ± 2.1%, 3.5±2.8 and 9.4±8.6, respectively. For LSRs, CSRs and RSRs, it was 6.8±7.3%, 6.4±8.3% and 11.8±5.5%.

Inter-observer variability for GLS, GCS and GRS was 7.1±5.1%, 8.5±8.9% and 11.8±6.9%. For LSRs, CSRs and RSRs, it was 10.7±5.0%, 7.7±5.8% and 12.6±10.5%.

## Discussion

LV remodeling after AMI is an importantly prognostic issue. Global longitudinal strain (GLS) by 2D speckle tracking echocardiography has proven to be predictive for LV remodeling but no reports have investigated its role in patient with preserved EF. We reported a new index, the injury longitudinal strain (InjLS), defined as the average strain of which segmental longitudinal strains >– 15%. Our study assessed the clinical value of LV deformation performance indices based on 2D speckle tracking echocardiography in predicting LV remodeling in patients with acute myocardial infarction with EF > 40%.

The well-known implications of LV remodeling include higher mortality and higher rates of heart failure, and the incidence is around 30%–35% [1–4]. In our study, LV remodeling was found in 28.4% of the cohort, and this occurrence rate is comparable to but lower than the rates reported in previous studies [1–4,12,14,16,22]. EF has been associated with LV remodeling [10,12,13]. The baseline LV systolic functions in our cohort were relatively well preserved, which could explain the lower rate of LV remodeling in our study.

### Two dimensional speckle-tracking echocardiography for remodeling

In 2008, Park et al [10] first reported the predictive value for LV remodeling of longitudinal strain measured by speckle tracking echocardiography at 7 LV segments of the left anterior descending coronary artery territory. Fifty patients with anterior-wall AMI were enrolled, and 22 patients developed LV remodeling. Previous studies discovered the value of GLS for predicting LV remodeling in STEMI or NSTEMI [11–13,16]. Bochenek et al evaluated 66 patients with ST–T elevation myocardial infarction treated by primary PCI [11]. D'Andrea et al evaluated 70 patients with recent NSTEMI [12]. Zaliaduonyte et al evaluated 82 patients within 48–72 h of the onset of AMI [13]. Lacalzada et al studied 97 patients with acute myocardial infarction treated with primary PCI [16]. However, the VALIANT Echo study demonstrated that circumferential strain rate was predictive for LV remodeling but that GLS and LSRs were not [17]. That study investigated 603 patients with LV dysfunction or heart failure after myocardial infarction. Only 311 cases had adequate image quality to permit assessment of all longitudinal and circumferential strain and strain rate. The GLS and LSRs were derived only from the mean value of apical 4- and 2-chamber views. Probably, the mean valve of a total of 12 segments was not representative of global values. A large study by Joyce et al reported an association between GLS and adverse LV dilatation after STEMI in 1041 patients [15]. The population was divided into 2 groups according to a median valve of GLS of –15.0%. Patients with baseline GLS greater than –15.0% exhibited greater LV dilatation at 3- and 6-month follow-ups compared with patients with GLS equal or less than -15.0%.

Our study is the only one that has assessed the clinical use of 2D speckle tracking echocardiography in patients with relatively preserved LVEF after AMI. In multivariate logistic regression, InjLS was an independent predictor for LV remodeling and indeed was more predictive than WMSI. GLS, GCS or GRS was not different between patients with or without LV remodeling. The association between GLS and infarct size has been studied recently [23–27]. Sjoli et al investigated GLS after revascularization in 39 patients with STEMI and treated with thrombolysis [26]. A cutoff value of –15.0% for GLS was precise to identify a large myocardial infarct. Gjesdal et al discovered that the GLS level identified by 2D speckle tracking echocardiography is closely related to myocardial infarct size as determined by contrast-enhanced magnetic resonance imaging during chronic ischemic heart disease [27]. A strain value of –15% has 83% sensitivity and 93% specificity at the global level and 76% sensitivity and 95% specificity at the territorial level to identify infarction. In our study, InjLS was defined as the average of segmental longitudinal strains > –15%. The number of injured segments between the non-remodeling and remodeling groups is no significant different. Possible because our patients have preserved EF and the infarcted area is relative small. We hypothesized the adverse remodeling depend on the severity of injury other than the extent of injured or infarcted area in patient with preserved EF. Other LV deformation indices are not predictive for several potential reasons: According to the previous study [28], the longitudinal strain that is predominantly governed by the subendocardial regions, is the most vulnerable and sensitive to the presence of myocardial disease. While mid-myocardial and epicardial regions contribute more to circumferential and radial strain. Circumferential or radial strain are relatively preserved or compensated to maintain the LV systolic function in the initial process. In the present study, we investigated patients with preserved EF, and thus the likely extent or severity of myocardial infarctions was probably modest. We suppose that circumferential and radial strains are relatively preserved. Second, the contractility of segments in the remote zone could compensate to maintain LV systolic function. Global LS, GCS, and GRS represent the global LV contractility and perhaps do not reflect the severity of the infarct area in a cohort with preserved EF. Our echography was performed after PCI, and the regional wall motion of the culprit territory could be improved by the intervention. Myocardial contractility recovery in STEMI can occur within 2 days, as demonstrated by Ingul et al [29]. Global LS is correlated with EF or WMSI [12]. Since our cohort had preserved EF, the infarct size could be smaller than that in previous studies. These potentially explain why GLS, GCS, GRS or EF is not identified as a predictor in our study. We assume abnormal segments play a more important role in adverse LV remodeling in patients with preserved EF. The present study demonstrated WMSI was a predictor in ROC analysis but InjLS is more predictive than WMSI in multi-variate logistic regression analysis. In subgroup analysis for STEMI, InjLS was also demonstrated as a unique predictor by multivariate logistic regression or ROC analysis.

### Clinical predictors of remodeling

In our study, peak CK-MB was an independent predictor of adverse LV remodeling, as previous studies reported [6,16,30]. The appearance of LAD as the culprit vessel was not associated with remodeling, even in subgroup analysis for STEMI. Potential explanations include the following: 68.7% of our population had multi-vessel disease, and LAD stenosis was found in 89.2% patients although it was not the culprit lesion. Further, the TIMI flow of the infarct-related artery after PCI was Grade 3 in all patients. STEMI had a tendency forward adverse LV remodeling, but this finding was not statistically significant because of the small group. In the NSTEMI group, only 5 cases had LV remodeling. In subgroup analysis, the door-to-balloon time was longer in remodeling group but the symptom-to-balloon time was not different. Probably because most of our patients live in the countryside and they take more time to arrive in our hospital, the symptom-to-balloon time was high.

### Limitations

The present study has several limitations. Our study cohort was relatively small and was limited to patients with few complications. Patients who required mechanical ventilation or intra-aortic balloon pumping were excluded because images with vivid endocardial edges through the whole cardiac cycle are required for 2D speckle tracking analysis. Our group was not limited to STEMI, and some patients with Killip class IV were also enrolled. However, we demonstrated the feasibility of the 2D speckle tracking technique in the real-world setting of acute coronary syndrome with patients who were not highly selected. We performed echocardiography after PCI (mean 2.7 ± 1.6 days after PCI), which is not like protocols of major published studies in which echocardiography was performed on the first day of admission or immediately after PCI. Recovery of myocardial contractility following ischemia or infarction after PCI can occur early [29]. Our study investigated patients with preserved EF, and the possible extent or severity of their myocardial infarctions probably were modest. In 5 patients, all segmental longitudinal strain was smaller than –15%. InjLS was available for 78 patients (94%). We did not evaluate the circumferential or radial strain of the injury segments for several reasons. There is rare research to investigate the relationship between infarction size and circumferential or radial strain. We lack the suggestion for cut-off value of circumferential or radial strain to define injury segments. Besides, as we mentioned earlier, circumferential or radial strain are relatively preserved or compensated to maintain the LV systolic function in the initial process since our cohort had preserved EF. And reproducibility of circumferential or radial strain is not as good as longitudinal strain.

We used CK-MB instead of troponin-I, because troponin-I is checked once only for diagnosis in our hospital and is not follow-up routinely. So we don’t have the peak value of troponin I. Serial CK-MB are collected once acute coronary syndrome is diagnosed.

The criteria of our government insurance for statin is LDL>100 mg/dL or total cholesterol >200 mg/dL. Only 30.5% patients meet the criteria (non-remodeling vs remodeling group: 17[28.8%] vs 8 [34.8%], P = 0.6). Then use of statin depended on physicians and patients’ decision when the government insurance did not cover the cost of statin. This could explain the low percentage of patients taking statins although about 80% patients have LDL>70 mg/dL in both groups (53[89.8%] vs 20 [87%]).

## Conclusions

LV remodeling occurred in 28.9% of AMI patients with preserved EF even after PCI. The 2D speckle tracking echocardiography was a promising, feasible, and noninvasive modality to evaluate myocardial deformation in this cohort. In the present study, InjLS was an independent predictor for LV remodeling in patients with preserved EF.

## Supporting Information

S1 Dataset The database of the present study. (XLSX)
